# Realization of Self Through Science

**DOI:** 10.15695/jstem/v9i1.08

**Published:** 2026-08-14

**Authors:** Michael Coe, Kristen Cram, Carolyn Hester, David Jones, Anna Kiley, Tony Ward

**Affiliations:** 1 Cedar Lake Research Group LLC; 2 School of Public and Community Health Sciences, University of Montana, Missoula, MT

**Keywords:** Attitudes and achievement, Evaluation and theory, Science education, Social development, Student beliefs and values

## Abstract

Science education programs are often designed and evaluated based on how they influence student attitudinal variables such as interest, motivation, aspirations, or self-efficacy that may mediate long-term attainment of degrees or careers in scientific fields. This study disaggregates student reports of how a science education program affected their interest in science and science careers in order to examine how gender and pre-existing attitudes toward science were related to their responses to program activities. No average program effect was found across all students, but there was clear heterogeneity of treatment effect based on gender, developmental interest trajectories related to science, and prior foreclosure of science and science career interest. Themes in student comments about program effects on their interest in science were related to reports of positive, negative, or no program impact, with different subgroups of students reporting different reasons for increases, decreases, or lack of change in their interest in science and science careers. These findings are discussed in relation to career counseling research and recent psychological and neuroscience research and theory focused on refining the definition and measurement of interest, understanding developmental processes involved in interest, and clarifying the relationship of interest to motivation, persistence, and achievement.

## INTRODUCTION

### Student Attitudes and Achievement in Practice and in Research.

Many approaches to promoting secondary school student science achievement have focused on the development of precursors that support the attainment of improved performance in science, technology, engineering, and mathematics (STEM) courses, degrees, jobs and careers. Student attitudinal variables such as STEM-specific interest, motivation to achieve, persistence, aspirations, and self-efficacy, or self-concept formulations such as scientific identity or scientific possible selves may help explain why some middle and high school students enroll in STEM courses in college, earn STEM degrees, or pursue STEM-related jobs or careers (e.g. Bandura, 1977, 1997; Bandura et al., 2001; Britner & Pajares, 2006; Kubsch et al., 2022; Pajares, 2005; Renninger & Hidi, 2019; Renninger et al., 2015a, 2015b; Schunk & Pajares, 2002; Watt, 2006; Zimmerman, 2000). The dynamics and functional roles of these psychological characteristics may differ based on gender (Häussler & Hoffmann, 2002; Renninger et al., 2015a); development of confidence and self-efficacy may be especially important for girls who contemplate entering STEM courses or careers, while boys may be more likely to have their pre-existing confidence, interest, and self-efficacy shaken by the challenge of advanced STEM coursework (Ashlock et al., 2022; Bench et al., 2015; Sakellariou & Fang, 2021). Both overconfidence and low confidence can have negative effects on educational aspiration and motivation (Pintrich, 2003; Vancouver & Kendal, 2006). Students may also respond differently to educational interventions aimed at leveraging self-efficacy or related constructs depending on their gender (Alhadabi, 2021; Heaverlo et al., 2013; Vincent-Ruz & Schunn, 2017, 2018; Zeldin & Pajares, 2000).

The bidirectional influences among self-efficacy, interest, goal-setting, knowledge and achievement can improve learning or alternatively can exacerbate learning difficulties (Linnenbrink-Garcia et al., 2011). Interest and self-efficacy can be mutually reinforcing, but low self-efficacy can lead to low interest and low goal-setting, overconfidence can lead to low study effort, failure to achieve an expected goal can lower self-efficacy and interest, etc. These dynamics argue for scaffolding learning and mastery experiences to create optimal educational trajectories for students that help them set and meet appropriately challenging goals, building their confidence, self-efficacy, and interest (Bandura, 1991; Bandura & Locke, 2003; Kubsch et al., 2022; Pintrich, 2003; Renninger & Hidi, 2016; Renninger & Su, 2019; Zimmerman, 2000).

The cultivation of positive attitudes and self-concepts related to STEM disciplines has become a key target outcome for science education programs. These factors are hypothesized to serve as causal mediators that drive science achievement; they also are proximal in time to middle school and high school educational interventions. Program designers are presented with a challenge: how to promote student engagement, interest, aspirations, and positive attitudes toward science when different progressions of experiences and supports are required for different students at different points along their developmental trajectories (Renninger & Hidi, 2016). For researchers, tracking long-term outcomes for large numbers of students can be expensive and technically difficult as they move from grade to grade and on to adult life. Measurement of attitudinal change soon after an educational experience or intervention is more feasible and can be well aligned with programs designed to influence attitudes. As a result, changes in student self-efficacy, interest, or similar constructs often serve as proxy measures of success for efforts to spur long-term growth in academic and career development by influencing student attitudes.

### Attitudes and Achievement in a Specific Science Education Program.

Program evaluation research conducted for the University of Montana’s Research Education on Air and Cardiovascular Health (REACH) program has used the paradigm outlined above. Successive iterations of the student-centered, inquiry-based program have sought to generate long-term student success in health science by improving student attitudes toward science learning, higher education, and jobs and careers. The program provides curriculum on air quality and health, low-cost air sampling equipment, and teacher support to facilitate student engagement in research practices focused on air pollutants and effects on human health. Over an academic year, high school and middle school students design and conduct research on local air quality and present their findings in local or regional settings, including a university-based research symposium. Beginning in the fall with curriculum modules on air quality measurement, impacts on human health, and fundamentals of research design and analysis, students work in small groups to design and conduct their own unique research projects to investigate factors that affect air quality in their homes, schools, or other local sites, capitalizing on student interest in place-based issues and careers that affect them, their family, and their community. Near the end of the year, they practice science communication by making presentations detailing their research motivation, hypotheses, design, analysis, and findings. Feedback from teachers and students has informed refinement of the program, which has grown in popularity among schools and teachers and generated enthusiastic testimonials and discussions, along with preliminary evidence of efficacy for student learning and effects on student attitudes (for more details on the program curriculum and previous findings see Coe et al., 2023; Delaloye et al., 2016; Delaloye et al., 2018; Kiley et al., 2022; Taylor et al., 2021; Ward et al., 2016).

Recent attempts to conduct quasi-experimental research for more robust estimation of program effects have been disappointing. The COVID-19 pandemic introduced unexpected challenges, including reduced sample sizes, unexpected programmatic changes from year to year, and increased variability in program implementation. Statistical estimates of average effects across large numbers of students have been difficult to implement in our geographically dispersed, low-population rural and small-town settings, and have not produced strong evidence of program effectiveness. Despite persistent teacher and school enthusiasm for the program and growing annual participation by schools that deem participation to be worthwhile for their students, a rigorously defined measurable impact on the average student has been elusive.

These results prompted us to reconsider the context within which high school science education practices are implemented and experienced and to ask some reframed evaluation questions. In this report, we briefly summarize quasi-experimental findings from student data collected during the 2021–2022 school year. Then we present a disaggregated qualitative analysis to illuminate why student subgroups reported contradictory program effects that were masked when aggregated into a single average treatment effect estimate for all students. Our analysis was based on questions about whether this program and others like it might be better understood by examining their potential effects on different types of students, in addition to the average effect on all students, and whether literature on student development might inform a more useful approach to program evaluation.

### What Counts as an Effect on Student Interest?

Two fundamental insights about the quasi-experimental findings informed the qualitative analysis we report here. First, a finding of no average effect across a large group of students does not necessarily mean there was no effect on particular subgroups. A program may have a positive effect on only certain students, or it may have a positive effect on some students and a negative effect on other students. Subgroup analysis based on demographic indicators may be fruitful in identifying different responses to an educational program (Schudde, 2018). However, groups based on demographic identity indicators may not be the only or best way to understand heterogenous treatment effects. There is a burgeoning literature on conceptualizing and analyzing heterogenous treatment effects in educational research, medicine, social science, political science, and other fields (e.g. Cintron et al., 2022; Schochet et al, 2014; Xie, 2013).

The second principle we applied was the idea that secondary school experiences are not just about learning academic content; these are crucial developmental years for student learning about themselves. Middle school, high school, and the years immediately after high school (college years for some) offer a buffet of life experiences for students to sample. This brings broadened opportunities to discover what they like and don’t like; what they are good at and what they are not so good at; what they want to do more of and what they want to do less of. Their courses and extracurricular activities broaden their understanding of options for pursuit of their hopes and dreams and for jobs and careers by offering samples of what life can be and what choices they can make. These are years in which students make great strides in discovering who they are. Experiences in a science education program may confirm their pre-existing orientation toward science or may alter their existing orientation in various ways—furthering their process of self-discovery, self-awareness, self-realization, and self-actualization.

Refinement of students’ awareness of their own aptitudes and interests can lead to improved educational outcomes; students who identify their career interests early may benefit from focusing and sequencing their course-taking and related preparation. According to recent data from the U.S. Department of Education, about a third of college students change their major within three years of enrollment and almost 10% change their major two or more times during their first three years of college (Leu, 2017). A recent poll of 817 U.S. college graduates found that while more than 82% consider college to be a good financial investment, 61% would change their majors if they could (Johnson, 2023). Of these, 26% would change their major in order to “pursue my passion” and 25% would change their major for “better/more job opportunities.” Other reasons cited were for better compensation or benefits (14%), to learn in-demand skills (14%), or because “I am personally dissatisfied with or stressed/burned out in my current career.” Johnson (2023) suggests that schools should “help students align their majors with their ideal career paths” and discusses the potential increase in cost for students who switch majors in terms of both time and tuition (Marcus, 2018). However, some studies have found that switching majors does not reduce college graduation rates or overall length of time in school if the change is made during the first four years of college; these metrics only decline if a change in major occurs outside the normal four-year college period, in the fifth year or beyond (Venit, 2016).

Career counseling in high school and later years attempts to help students better understand their aptitudes and interest in order to help them focus on pursuits and careers that will be well suited to them. In this arena and in the broader literature on adolescent development, there is a line of theory and research on “career foreclosure” which is often framed as a problem: students may fixate on specific career choices (or other aspects of identity) early, and thereafter fail to consider other possibilities that might be a better fit (Shaffer & Zalewski, 2011, p.62):

Foreclosure students have prematurely committed themselves to academic majors and future careers, but present themselves to academic advisors as very decided. From a developmental theory perspective, foreclosures represent an immature identity status and include students whose failure to explore their personal and vocational identities and achieve a mature identity threatens their academic success.

Writing about adolescent identity development in general, Marcia (1966; 1993; Marcia & Adelson, 1980) proposes that foreclosure status is when a commitment is made without exploring alternatives. His four categories of late adolescent “identity statuses,” including foreclosure along with identity diffusion, moratorium, and identity achievement, have been well supported in subsequent research (e.g. Meeus, 2011; Meeus et al., 2012). Meeus and Crocetti (Crocetti et al., 2008; Meeus et al., 2010) extend this model by elucidating the importance for adolescent identity formation of “identity status trajectories” measured along three dimensions:

**Commitment** *refers to strong choices that adolescents have made with regard to various developmental domains, along with the self-confidence that they derive from these choices*. **In-depth exploration** *represents the ways in which adolescents maintain their present commitments. It refers to the extent to which adolescents actively explore the commitments that they already have made by reflecting on their choices, searching for information about these commitments, and talking with others about them*. **Reconsideration of commitment** *refers to the willingness to discard one’s commitments and to search for new commitments. Reconsideration refers to the comparison of present commitments with possible alternative commitments when the present ones are no longer satisfactory. The Meeus-Crocetti model assumes that identity is formed in a process of continuous interplay between commitment, in-depth exploration, and reconsideration.”*

(Meeus et al., 2012, p.1009).

These concerns about the potential dangers of early career foreclosure and the importance of in-depth exploration and reconsideration of commitment differ from the perspectives of others who emphasize the potential benefit of early commitment, focused dedication, and the accumulation of skill-building practice through limitations on distraction. This perspective was popularized by Malcolm Gladwell’s argument for the importance of 10,000 hours of practice for achieving success in sports, computer science, business, music, physics, engineering, and mathematics (Gladwell, 2008). The so-called “10,000-Hour Rule” was inspired in part by the work of Ericsson on the acquisition of expert-level skills (Ericsson et al., 1993, 2007) but has been disputed by others who claim that accumulation of practice accounts for less than 30% of variation in expert skills across various domains of expertise (e.g., MacNamara et al., 2014). A similar perspective, widely popularized as the “5/25 Rule” for personal success and attributed (possibly apocryphally) to investor Warren Buffet, recommends making of list of one’s top 25 goals, then “avoiding at all cost” the bottom 20 in favor of focusing on the top five goals until they are achieved.

Understanding links between the development of identity and sense of self, student educational and career choices, the development of expertise in specific fields, career success, and a sense of meaningfulness in work (Martela & Pessi, 2018) is a matter of intense interest to those who aim to assist adolescents in their pursuit of education and a fulfilling life. Educational interventions should perhaps be evaluated, in part, for how they assist students in unraveling the puzzle of their own aptitudes and “possible selves” (Delaloye et al., 2018; Markus & Nurius, 1986; Packard & Nguyen, 2003), in addition to more traditional research examining group-based linear growth in average student interest or achievement in a particular domain.

Recent developments in interest theory bring together these themes and help delineate what we mean by “interest.” By understanding the neurological foundations of interest and attending to how interest can be understood, supported and shaped as it develops over time, rather than being conceptualized as a fleeting, momentary feeling or an unchanging, dispositional personality characteristic (Hidi & Renninger, 2006; Renninger & Hidi, 2016, 2021), programs can better serve specific student learning needs. Students in different phases of interest in a topic respond differently to educational interventions that are designed to trigger or enhance their interest, efficacy, aspirations and learning. Experiences that trigger early phases of interest may not work well to maintain or expand the already well-developed interest of some students. Conversely, activities that maintain or expand the more well-developed interest of other students may not be effective in sparking initial interest among those who have yet to become interested. Educators and researchers who are “flying blind” without attending to these developmental trajectories are unlikely to optimize program design or evaluation research for programs that focus on interest, efficacy and related constructs as key targets and measured outcomes.

Armed with these perspectives, we attempted to identify and understand subgroups of students that demonstrated different patterns of response to the REACH curriculum—groups that may be replicable in other secondary school student populations and that may be useful to consider in science education program design and evaluation.

## METHODS

### Study Overview.

With funding provided by the National Institutes of Health (NIH) Science Education Partnership Award (SEPA) program, REACH provides professional development, curricula, and equipment to high school classrooms located in rural areas of Montana, Idaho and Alaska. Teachers implement the program in their science classes, enabling students to design their own research projects, collect data, analyze results, and present their findings related to indoor air pollution and potential resultant impacts to human health. The REACH program began in 2003, and engages over 1,000 students in nearly 50 classrooms each year.

Evaluation research for the program used a mixed-methods design focused primarily on survey data. Data collection methods were reviewed by the University of Montana Institutional Review Board which designated the research as exempt from further review and waived documentation of informed consent (IRB #155–18). The surveys included fixed-response and open-ended questions related to student interest in science learning and careers, feedback on program components and their perceived impact, and recommendations for improvement. Comparison group student survey data was collected from available comparison classrooms to provide context. Data were analyzed separately by school year because COVID-19 disruptions and related school accommodations had a strong potential for affecting outcomes.

The data reported here come from survey responses collected from students during the 2021–2022 school year, when COVID-19-related school closures and emergency remote learning accommodations had subsided amid a return to routine classroom practices. The focus of these survey questions was student interest in further science learning, their aspirations for science degrees, jobs or careers, and their experiences in the REACH program. Questions on science interest and aspirations were included on surveys for treatment and comparison students; questions about the program were only asked of program participants. Available logistics and institutional considerations precluded random assignment or close matching of comparison group classrooms with the participating program (treatment) classrooms. Instead, the comparison group included a convenience sample based on nearby schools and teachers willing to assign students in similar science or math classes to participate in the surveys.

### Student Surveys.

Students were asked to complete online pre-surveys near the beginning of the school year and again near the end of the school year. Individual pre- and post-survey responses were matched for those students who responded at both times. The surveys were developed to align with NIH-SEPA programmatic goals and with the science curriculum focus of the REACH program in particular; some questions were adapted from published surveys such as the STEM-CIS (Kier et al., 2014) and the STEM Career Interest Questionnaire (Tyler-Wood et al., 2010). In addition to general sets of fixed-response and open-ended questions about interest in science learning, science degrees, and science jobs and careers, the surveys asked specifically about interest in health science learning, health science degrees, and health science careers. The fixed-response survey modules included sets of questions that could be examined individually or combined into scale scores for analysis. Post-surveys included direct questions about the perceived influence of the program on students’ science interest and aspirations. Specific wording of survey questions was updated over the years of the project; examples of the wording used in this study (or more current versions) are available from the corresponding author.

### Data Analysis.

Scale scores developed from sets of fixed-response questions were analyzed using standard descriptive statistics to summarize the distribution of responses along with ordinary least squares and multilevel regression modeling to estimate observed differences between the treatment and comparison groups. Narrative responses to open-ended questions were analyzed using a structured process for identifying and quantifying themes in the responses and then determining the distribution of those response themes across subgroups of students.

Some fixed-response questions were used to define student categories for subgroup analysis. Scale scores for sets of fixed-response questions on student interests and aspirations were developed by calculating a mean response score across each set of questions. Group differences in post-survey scale scores were modeled using classroom group (treatment vs. comparison classrooms) and individual student pre-survey scale scores on the same set of questions. Potential gender and age effects were also tested as model covariates.

For the narrative responses to open-ended questions, thematic analysis (Braun & Clarke, 2006, 2019) was first used by a panel of three judges to identify common themes in the student comments and response patterns. Each judge reviewed the individual student responses a total of three times. First, each reviewer independently identified potential themes in the student comments in the context of their responses to baseline and follow-up fixed-response science interest items. The panel then met and agreed upon a shared framework for defining and coding common themes. During a second independent reading, each reviewer applied this shared coding framework to each of the student responses. Finally, reviewers met to resolve any discrepancies in how they had defined and applied the common themes and to reach consensus on final coding of specific student responses. A single student’s responses could be assigned more than one thematic code to describe their science interest profile. Subgroups of students were then analyzed to determine how likely each group was to report each of the themes in their responses to questions about science interest, science degree and career aspirations, and how their program experiences had affected their interest and aspirations.

## RESULTS

### Sample.

The sample for this study included 413 students in the 2021–2022 school year who responded to both pre- and post-surveys, yielding individually matched pre- and post-survey responses. The 254 treatment group participants were located in 14 high schools, with an average of 18 student responses per school. Their survey responses regarding program impact were further examined for potential interest trajectory subgroups. Of these students, 26.8% were in grade 9, 27.6% in grade 10, 37.8% in grade 11, and 7.9% in grade 12. More than half (51.6%) reported their gender as female, while 42.1% identified as male and 6.3% marked “prefer not to answer.” The 159 comparison group students were located in three high schools with an average of 53 student responses per school. Students were in grade 9 (29.6%), grade 10 (36.2%), grade 11 (26.9%) and grade 12 (7.3%). They reported their gender as female (50.4%), male (44.1%), or “prefer not to answer” (5.6%).

### Observed Differences Between Treatment and Comparison Groups.

The treatment and comparison groups were not randomly assigned or chosen through a process designed to ensure matched characteristics and therefore were not equivalent at baseline. The comparison group was significantly younger on average at the beginning of the year (mean age 15.0 years old, SD = .91 vs. 15.5 years old, SD = 1.04 for the treatment group) and began the year with significantly lower interest in science, as well as marginally lower interest in science education and science careers. Comparison group students were more likely to be enrolled in lower grade required courses than treatment group students, who were more likely to be enrolled in higher grade elective courses. Strong causal inferences could not be drawn from comparisons of the two groups and were not emphasized in subsequent analyses or in this report. However, analyses were conducted to explore descriptive findings and to contextualize exploration of student comments among subgroups within the program participants.

Ordinary least squares and multilevel statistical models were estimated to determine if treatment group membership or baseline variables predicted outcomes of interest, including interest in science learning, interest in science education (degree attainment), and science jobs or careers. In each case, baseline levels of interest predicted end-of-year interest, but group membership, gender, and age were not significant predictors of outcomes after accounting for baseline interest. In general, the consistent finding across these analyses (not reported in detail here) was that the comparison group began and ended the year with lower interest in science learning, science education, and science careers compared to the treatment group, and that there was no detectable average effect of program participation. When each group was analyzed separately, the predominant finding was that initial interest levels strongly predicted students’ end-of-year interest levels, but there was no average gain in interest over the course of the year.

However, these null findings were not the end of the analytic road, since a lack of an average effect can mask subgroup effects and exploratory findings of interest. The next phase of analysis explored individual science interest trajectories and student reports about how their program experiences influenced, or did not influence, development of their science interests.

### Subgroup Analysis Based on Reported Interest Trajectories.

In order to investigate possible heterogenous treatment effects, students in the treatment group were classified into groups based on their post-survey responses to questions about how the program affected their interest in science and science jobs or careers. On a five-point fixed response scale, those who reported that the program made them “a lot more interested” or “a little more interested” in science were classified into a group that reported that the program made them more interested in science. Those who reported that the program made them “much less interested” or “a little less interested” in science were classified into a group that reported that the program made them less interested in science. Students who reported that the program “didn’t really change my level of interest in science” made up the remaining group. A total of 186 students answered this fixed-response question and also identified as male or female (students who preferred not to answer the gender question were too few to analyze as a separate group). See Table 1 for the proportion of respondents in each resulting interest-trajectory subgroup, along with other subgroup characteristics.

To check the validity of the classifications, differences between pre- and post-survey ratings of science interest were compared across the three groups that had been created based on self-reported program impact. Table 1 includes the mean scores of each group on a baseline science interest scale administered at the beginning of the school year along with group mean scores on a retrospective pretest version of the baseline scale and a follow-up science interest scale, both administered at the end of the year. Students who reported positive program impact (the program made them more interested in science) also separately reported greater average interest in science at the end of the year than they had reported at baseline or on the retrospective pretest. Students who reported that the program didn’t affect their interest in science (no program impact) and those who said the program made them less interested in science (negative program impact) showed no change on average in science interest at the end the year.

More than half of all students (51.1%) reported that the program had no effect on their interest in science topics; 37.6% reported that the program made them more interested in science topics, and 11.3% reported that the program made them less interested. Girls were significantly more likely than boys to report that REACH made them more interested in science topics; conversely, boys were more likely than girls to report that the program made them less interested in science (see proportions in lines two and three of Table 1; χ^2^ (2, N=186) = 7.23, *p* < .05). This difference was not explained by age differences; boys and girls did not differ significantly in average age, with girls averaging age 15.50 and boys averaging age 15.46 at the end of the year.

In order to better understand these subgroups and their response to treatment, we studied their comments about why or how the program made them more or less interested in science or did not affect their interest. Responses to two prompts were considered: “Please comment on how the program affected your interest in science” and “Please comment on how the program affected your interest in having a job or career in science.” Thematic analysis of these comments in the context of responses to baseline and follow-up fixed-response science and science career interest questions yielded 10 themes found to be common across multiple student responses. These are presented in Table 2 along with example student comments and the proportion of students whose responses were coded into each theme.

Of the 131 students who provided comments that were classified into at least one theme, 40% (*n* = 53) were male and 60% (*n* = 78) were female. The number of students whose comments were classified into a specific theme varied from 4 to 30 individuals. Within each theme classification, sample sizes limited statistical power (sensitivity) for detecting gender differences; for each theme considered separately, differences in the proportions of boys and girls who made comments that were classified into each theme were not statistically significant (Table 3). However, the overall pattern of theme classification by gender may provide insight into the finding (above) that girls were significantly more likely than boys to report that the program made them more interested in science topics. Themes 6 – 10 denote specific program components that students found interesting. Each of these five themes was reported by a higher percentage of girls than boys, a pattern that is statistically significant (*p* < .05), with a binomial probability of 0.03, akin to the likelihood of flipping a coin five times and obtaining 5 heads.

Theme 6, “learning about research methods and doing research was interesting,” was indicated by comments from 11.4% of students (11.3% of boys and 11.5% of girls); among those students whose comments were classified into this theme, 60% were girls. Theme 7, “learning about air quality science content was interesting,” was indicated by comments from 16.8% of students (11.3% of boys and 20.5% of girls); among those students whose comments were classified into this theme, 73% were girls. Theme 8, “science and research are more fun and interesting than I realized,” was indicated by comments from 18.3% of students (13.2% of boys and 21.8% of girls); among those students whose comments were classified into this theme, 71% were girls. Theme 9, “seeing what scientists do made me excited to help people in a career that is relevant,” was indicated by comments from 16.8% of students (15.1% of boys and 17.9% of girls); among those students whose comments were classified into this theme, 64% were girls. Theme 10, “presenting projects and getting feedback on my work was interesting,” was indicated by comments from 3.1% of students (1.9% of boys and 3.8% of girls); among those students whose comments were classified into this theme, 75% were girls.

## DISCUSSION

Since at least the Sputnik crisis of the late 1950s, the U.S. public education sector has focused enormous effort and budget resources on cultivating student skills and careers in the STEM fields of science, technology, engineering and mathematics (Wissehr et al., 2011). Much of this energy has been directed toward enhancing the STEM interest and aspirations of high school and middle school students, in part because student choices starting in middle school determine whether they take the prerequisite secondary school courses and course sequences necessary to prepare for STEM college degrees and careers. This national emphasis on STEM education may be viewed in part as an effort to ensure that all students have an opportunity to participate in STEM fields by reducing barriers to participation and providing support for previously underrepresented groups.

Evaluation research aimed at assessing the value and success of these efforts has often looked for an overall increase in the number of students who pursue further education and careers in STEM fields, along with increased proportions of women and other demographic groups historically underrepresented in these disciplines. In much of this research, predictors or proximal indicators of these longer-term career development goals have served as proxy outcome measures for evaluating the hoped-for eventual impact of programs aimed at growing the U.S. STEM workforce. These more easily measured near-term outcomes include behavioral indicators—grades, test scores, and course-taking—as well as psychological indicators such as interest in science generally or in particular STEM topics or fields, aspirations for college degrees or careers in STEM disciplines, motivation, self-efficacy, or self-concept schemas, such as science-oriented components of personal identity or envisioned future personal possibilities.

Converging literature from interest theory, career and vocational counseling, and adolescent identity development research may inform greater specificity in how STEM educators approach their goal of helping students find their way into STEM majors and careers or, at least, into greater appreciation of how science literacy can enhance adjacent careers and life pursuits. Although early interest surveys were created in the service of providing vocational counseling, until recently much of this work treated interest as a fixed characteristic rather than something that develops and can be stimulated, cultivated and shaped (Renninger & Hidi, 2016).

The data reported here suggests that design and evaluation of these kinds of programs would benefit from a more fine-grained attention to individual variation and, in particular, to the developmental trajectories of students’ psychological orientation toward science education and career-building. Although the findings are based on student self-reports, the validity of the self-reported program impact classifications is supported by correlation with observed patterns of baseline and follow-up reports of science interest. While more than a third of students reported a positive program impact, more than half reported no program impact, and there was no detectable growth in overall average science interest. There were, however, potentially meaningful differences among students who reported no program impact. These appear to be related to career foreclosure: pre-existing attitudes toward science and science careers that were already firmly positive or negative by the time of program participation. Students who are already committed to a science career, and who may have already settled on a particular STEM topic or discipline, present different challenges and opportunities to educators than students who have already strongly ruled out further science education or career interests.

Further, among students who reported a positive program impact when asked directly about how the program influenced their interest, a more detailed examination of their comments about the program revealed specific program activities or emphases that elicited greater interest for particular subgroups, along with potential gender differences in these dynamics. Interest in science among these students as measured by other questions was in fact higher on average at the end of their program participation than at baseline. While 43% of this group found that “science and research were more fun and interesting than I realized,” almost 40% specifically reported that “learning about air quality science content was interesting,” and almost 40% reported that “seeing what scientists do made me excited to help people in a career that is relevant.” More than a quarter of the group that reported positive program impact found learning about research methods and actually doing research to be interesting. There was some overlap, but these were distinct groups; many students were interested in the air quality and health science content but not in research methods, or vice versa; some students were particularly interested in the relevance of making a positive contribution to their community.

Girls were more likely than boys to report that the REACH program experience made them more interested in science; girls were less likely than boys to report that the program decreased their interest. Comments from girls were more likely to indicate specific avenues through which their interest was increased, notably that “learning about air quality science content was interesting” and “science and research are more fun and interesting than I realized.” These findings may be interpreted to reflect a softening of pre-existing career foreclosure as well as a weakening of perceived barriers to girls’ entry into STEM disciplines. This interpretation assumes that girls are more likely than boys to have made an early decision that science is not for them, and conversely, that boys are more likely to have made an early decision that science is for them. In either type of foreclosure, actual exposure to “doing science” may have triggered a reconsideration of these pre-existing attitudes and expectations and a greater willingness to explore intellectual, educational and career options—to discover and realize a self that students may not have previously thought possible.

Among those students who reported a negative program impact, a common theme was that doing scientific research is “too much work, too difficult, not fun.” For some, this may reflect an overconfidence among students who thought science would be easy for them and who were surprised to find that more work was required than they expected (Ashlock et al., 2022; Bench et al., 2015; Sakellariou & Fang, 2021; Vancouver & Kendal, 2006).

These findings can inform future program design and evaluation that is more responsive to variation in students’ pre-existing attitudes, expectations, individual needs, motivations, interests, and interest levels, phases or trajectories. Science education programs aiming to increase student conceptual understanding of science content and student participation in science degrees and careers could also benefit from application of related findings from career counseling research (e.g. Meeus et al., 2010, 2012), along with greater efforts to assess and respond to baseline levels of interest in science, or attitudes toward science, as they develop and deliver program activities. Design and evaluation of STEM education programs could also be improved through consideration of developments in interest theory, with a focus on triggering and supporting students’ interest in particular STEM fields and their applications that is based on assessment of particular students’ level or phase of interest in program content (Hidi & Renninger, 2006; Renninger & Hidi, 2020; Renninger & Su, 2019). Research and theory on interest development in relation to career development shows linkages between interest trajectories and “career decidedness,” finding that “most students who were studying science in university had a well-developed interest that had motivated their choice of programme, and their subject interest and career decidedness were linked” (Quinlan & Renninger, 2022, p. 863).

Students in this study reported different types and trajectories of interest in the science curriculum they were offered. Some were interested in applying knowledge of air quality and health to help people in their community; some were intrigued by learning to do rigorous research; some were interested in communicating findings to audiences; some were surprised to find that “doing science” was more fun or less difficult than they imagined, which increased their interest in pursuing more science learning. These findings are consistent with the view that interest is “a cognitive and affective motivational variable that develops and can be supported to develop” through the “triggering of interest in both earlier and later phases of interest” as well as activities aimed at “maintaining interest once it has been triggered” (Renninger & Su, 2019, p.167).

Interest can become self-rewarding. Initial interest may come from novelty or the surprising or entertaining aspects of a new topic, while later interest may be sustained and furthered by the satisfaction of developing skills and knowledge. Neurological research shows that as interest progresses to higher levels, the search for information, answers and solutions to problems in an area of interest is inherently rewarding; the pursuit of an interest creates more interest (Gottlieb et al., 2013; Renninger & Hidi, 2016). Educational activities that build on existing interest, knowledge and skills by “snowballing” student enjoyment of their own budding expertise will differ in important ways from initial activities that open students’ eyes to the possibility of interest in a new topic in which they have no existing expertise. Program design for science education should therefore include distinct activities that can trigger, maintain and support different phases of interest along with interest in different types of scientific work.

Curriculum designed with content and activities that address student needs at multiple points along possible interest trajectories can include both individual and team activities that maximize potential for individualized learning. By beginning STEM courses or programs with assessments and discussions of interests, expectations, and attitudes, educators can help students identify and reconsider pre-existing attitudes or stereotypes. By offering different activities to students with different baseline interest levels or attitudes, programs can nurture and support pre-existing interests whether these are untapped, partially formed, or already well developed.

In order to reach the kinds of student subgroups identified in this research, programs could more clearly include content designed to combat common negative attitudes or begin with early demonstrations of how science is used to help solve real-world problems of interest to students, before proceeding to skill-building and content knowledge acquisition. More than forty percent of students in this study reported that science and research were more fun than they had realized. Almost forty percent made comments indicating excitement at seeing what scientists do to help people in a career that is relevant. These comments address an initial awakening of interest and a reconsideration of foreclosed career paths. Grounding science content and science methods within a context of solving problems that students consider relevant and important may draw students in despite negative preconceptions about science, science careers, or about their own aptitude for doing science. As noted above, this approach appears to be especially important for supporting girls’ interest in science (e.g., Häussler & Hoffmann, 2002).

A more responsive, student-focused design might offer different roles in group research projects so students can focus on roles that are most appealing to them. Some students may prefer to take the lead on reviewing literature and summarizing what is already known about a problem or question, some may want to take the lead on designing and implementing quantitative methods, some may excel at writing or presenting findings. These different kinds of scientific work were called out by subgroups of students as particularly interesting. Programs could provide options to change roles for different projects, or to specialize in one type of science activity. This ensemble approach would offer students experience working in teams according to their aptitudes, collaborating with others who can make different contributions–which is how higher-level STEM projects and workplaces are increasingly organized.

In this research, additional subgroups of students reported interest in aspects of science other than air quality research, or being interested in science but not in a science career. Programs should include better options for students with these attitude profiles by offering a variety of ways to connect the focal topic of a science education program to other adjacent STEM disciplines or other academic, life and career interests. In addition to matriculation into college degrees in science disciplines, important program impacts may be found in adjacent areas, such as valuing and participating in citizen science projects, personal and community application of scientific findings, and other outcomes beyond traditional science careers.

## CONCLUSION

A recent review of experimental studies in political science found that although researchers are increasingly likely to publish non-significant findings showing no detectable effect for treatments, it is often difficult to tell whether these are “true, versus erroneous, nonsignificant results” (Kane, 2024, p.110). The author outlines seven possible explanations for non-significant findings, one of which is “countervailing treatment effects,” through which a treatment effect “can be near zero because treatment effects occur in opposite directions for different subgroups” (Kane, 2024, p.118). He recommends theorizing “about such heterogenous effects a priori (in the design stage)” (Kane, 2024, p.119) and including measures of potential moderating variables to formally explore how the treatment effect may vary across subgroups. Similarly, a recent special issue of *Educational Researcher* (Herrington & Maynard, 2019) focused on how to better understand null findings in experimental research and emphasized, among other methodological and contextual issues, “the importance of exploring variation in impacts” (Jacob et al., 2019, p.587). Recommendations include specifying whether the theory of change for a program or intervention suggests that “the program may be more effective for some participants or in some contexts” and designing evaluation studies accordingly (Jacob et al., 2019, p.582; see also Schochet et al., 2014).

The data we report here fit the above scenario in that we report a post-hoc examination of factors related to student interest trajectories that appear to influence program outcomes and explain previous null findings. We plan to include a priori measures and hypotheses about these factors in studies of a new program adaptation which emphasizes community-focused student research on the growing impact of extreme heat events and wildfire smoke. These studies will include more specific measures of interest trajectories prior to program participation as well as more sensitive outcome measures designed to detect heterogenous outcomes across heterogenous subgroups.

These findings have potential utility for better understanding the psychological and academic developmental trajectories of high school students with a particular focus on science and other STEM interests and how these may be influenced by educational programs. A better understanding of student interest profiles and trajectories is likely to be useful for designing program activities that respond to and build upon individual and subgroup differences. Measurement and analysis of program effectiveness is also likely to be improved by taking student interest trajectories and profiles into consideration and by designing studies to account for potential systematic heterogeneity of treatment outcomes. By integrating a broader theoretical basis and incorporating insights from related fields of study, program design and evaluation of STEM education programs can be improved.

REACH and programs like it are important because they present students with opportunities for self-discovery while offering hands-on experience with relevant science content and skills. These experiences may help students re-evaluate their assumptions about academic disciplines, career options, and their own interests and aptitudes. This may help them develop a personal and career trajectory that is informed by experience rather than prejudice, misconceptions, the opinions of others, or other roots of career foreclosure. Programs that provide hands-on experience in doing science may be helpful for different students in different ways, for example:

- Students may discover that science (or a particular science discipline, skill or activity) is more interesting to them than they previously thought; i.e., a better fit for their talents, aptitude, and interests than they realized.
- Students may discover that science (or a particular science discipline, skill or activity) is less interesting to them than they previously thought; i.e., not as good a fit for their talents, aptitude, and interests as they expected.
- Students may affirm their prior ideas about their interest, talent, or aptitude in science.
- Students may find that they have an interest in particular scientific disciplines or topics that are different from those they were previously aware of.
- Students may find that science is more difficult than they realized and that if they want to pursue STEM career aspirations they will need to work harder than they expected.

In any of these scenarios, students will have learned something about science and about themselves that will be useful in making informed choices about their path forward.

## Figures and Tables

**Table 1.** Student Subgroups Based on Self-Reported Program Impact on Science Interest.

	Student Subgroup		
	Less Interested	No Change	More Interested
Percent of Total Sample (N = 186)	11.3 %	51.1 %	37.6 %
Percent of Girls (n = 103)	5.8 %	52.4 %	41.7 %
Percent of Boys (n = 83)	18.1 %	49.4 %	32.5 %
Baseline Science Interest Scale Score	1.87 (.74)	2.62 (1.13)	2.80 (.91)
Mean (SD)			
Retrospective Pretest Science Interest Scale Score	1.84 (.79)	2.57 (1.14)	2.68 (.92)
Mean (SD)			
Follow-up Science Interest Scale Score	1.89 (.90)	2.59 (1.23)	3.13 (1.06)
Mean (SD)			
Science Interest Scale Score	.02 (.22)	−.03 (.09)	.33** (.11)
Mean Difference Baseline to Follow-up (SE)			
Science Interest Scale Score	.05 (.17)	.02 (.06)	.45*** (.10)
Mean Difference Retrospective Pretest to Follow-up (SE)			

Note.

** p <.01,

*** p <.001.

**Table 2.** Themes in Student Comments About Program Effects on Their Interest in Science.

	Theme (number of students)	Example comments	Percent of Decreased Interest Group	Percent of Unchanged Interest Group	Percent of Increased Interest Group
1	Too much work, too difficult, not fun (6)	*It makes me want to not go into a science career because it is too much work. Collecting data and creating graphs isn’t fun*.	41.7	1.6	-
2	Low initial interest was not changed by program (30)	*I just don’t like science. Still don’t really want to be into science*.	16.7	44.4	-
3	Not interested in air quality, interested in other aspects of science (18)	*It didn’t affect it much since I am interested in different types of science. It didn’t affect my interest, I thought it was cool but am interested in other fields of science*.	16.7	19.0	7.1
4	Interested in science topics but not a science degree or career (16)	*I’ve always viewed science as important and interesting but I’ve not ever had an interest in pursuing a career in science and the program has not changed that*.	-	12.7	14.3
5	High initial interest was not changed by program (18)	*I am still interested in science, especially biomedical science. Doing the REACH project didn’t really add or take away from my interest*.	-	28.6	-
6	Learning about research methods and doing research was interesting (15)	*Learning about how to correctly collect data and perform experiments was very interesting to me. REACH has helped me affirm my interests in science and I hope to pursue a degree in science one day, specifically biology and medicine*.	-	-	26.8
7	Learning about air quality science content was interesting (22)	*I thought it was very interesting how many different variables there are that can affect the air quality and also what we can do to prevent the air quality from getting worse*.	-	-	39.3
8	Science and research were more fun and interesting than I realized (24)	*It was fascinating to me to be able to pick what I wanted and research it. To see and analyze that data and make sense of something I was interested in sparked my interest and want to do it more*.	-	-	42.9
9	Seeing what scientists do made me excited to help people in a career that is relevant (22)	*It was interesting to see how careers in science can affect other people. I really liked it, and it made me more interested in our environment and the effects it has on different communities*.	-	-	39.3
10	Presenting projects, getting feedback on my work was interesting (4)	*It put into perspective how science research is usually presented and judged, allowing me to better understand what to prepare for in my future as a scientist. It let me get higher level feedback too which I think was cool to see people in that higher level judge our work*.	-	-	7.1

*Note*. N *= 131 students whose comments were classified into at least one theme. Some students returned multiple comments that were classified into more than one theme. Percentages indicate the proportion of students whose comments were coded into each theme. Comments from three students in the Decreased Interest group were not classifiable into any of these themes. The* Decreased Interest *group included 12 students, the* Unchanged Interest *group included 63 students, and the* Increased Interest *group included 56 students*.

**Table 3.** Themes in Student Comments About Program Effects on Their Interest in Science.

	Theme	Percent and (*n*) of Boys Classified Into Theme	Percent and (*n*) of Girls Classified Into Theme	Percent and (*n*) of Total Sample Classified Into Theme	Ratio of Boys : Girls Within Theme
1	Too much work, too difficult, not fun	5.7 (3)	3.8 (3)	4.6 (6)	50 : 50
2	Low initial interest was not changed by program	26.4 (14)	20.5 (16)	22.9 (30)	47 : 53
3	Not interested in air quality, interested in other aspects of science	9.4 (5)	16.7 (13)	13.7 (18)	28 : 72
4	Interested in science topics but not a science degree or career	13.2 (7)	11.5 (9)	12.2 (16)	44 : 56
5	High initial interest not changed by program	13.2 (7)	14.1 (11)	13.7 (18)	39 : 61
6	Learning about research methods and doing research was interesting	11.3 (6)	11.5 (9)	11.4 (15)	40 : 60
7	Learning about air quality science content was interesting	11.3 (6)	20.5 (16)	16.8 (22)	27 : 73
8	Science and research were more fun and interesting than I realized	13.2 (7)	21.8 (17)	18.3 (24)	29 : 71
9	Seeing what scientists do made me excited to help people in a career that is relevant	15.1 (8)	17.9 (14)	16.8 (22)	36 : 64
10	Presenting projects, getting feedback on my work was interesting	1.9 (1)	3.8 (3)	3.1 (4)	25 : 75

*Note*. N = *131 students whose comments were classified into at least one theme (53 male, 78 female). Some students returned multiple comments that were classified into more than one theme. Percentages indicate the proportion of students whose comments were coded into each theme. Differences between boys and girls were not statistically significant (Chi-square tests p > .10 in all cases)*.

## ABBREVIATIONS

NIH: National Institutes of Health
REACH: Research Education on Air and Cardiovascular Health
SEPA: Science Education Partnership Award
STEM: Science, Technology, Engineering, and Mathematics
